# Large deletion causing von Hippel-Lindau disease and hereditary breast cancer syndrome

**DOI:** 10.1186/1897-4287-12-16

**Published:** 2014-06-18

**Authors:** Karol Krzystolik, Anna Jakubowska, Jacek Gronwald, Maciej R Krawczyński, Monika Drobek-Słowik, Leszek Sagan, Leszek Cyryłowski, Wojciech Lubiński, Jan Lubiński, Cezary Cybulski

**Affiliations:** 1 Department of Ophthalmology, Pomeranian Medical University (PUM), Szczecin, Poland; 2 International Hereditary Cancer Center, Department of Genetics, Pathology PUM, Szczecin, Poland; 3 Department of Medical Genetics, Poznan University of Medical Sciences, Poznan, Poland; 4 Department of Neurosurgery, PUM, Szczecin, Poland; 5 Department of Radiology, PUM, Szczecin, Poland

## Abstract

Patients with intragenic mutations of the VHL gene have a typical disease presentation. However in cases of large *VHL* gene deletions which involve other genes in the proximity of the *VHL* gene a presentation of the disease can be different.

To investigate whether large *VHL* deletions that remove the *FANCD2* gene have an effect on the disease phenotype, we studied a family with a 50 kb large deletion encompassing these two genes. Four patients in this family were affected by VHL-related lesions. However one carrier of the deletion also had bilateral ductal breast cancer at age 46 and 49. Both tumors were of ~2 cm in diameter. On one side lymph nodes were affected. One tumor was ER- and PR-negative (HER2 s unknown) and the second was ER- and PR-positive, and HER2-negative.

Our study suggests that a deletion of *FANCD2* gene, an important gene in the DNA repair pathway, may be associated with an increased risk of breast cancer, but further studies are needed in this regard.

## Introduction

Von Hippel-Lindau (VHL) disease is a rare autosomal dominant disorder characterized by a predisposition to haemangioblastomas of the central nervous system (cHAB) and retina (rHAB), renal cell carcinomas (RCC), pheochromocytomas and paragangliomas, endolymphatic sac tumors (ELST), pancreatic neuroendocrine tumors (PNET), papillary cystadenomas of epididymis, and adnexal papillary tumors of probable mesonephric origin (APMO) [1-3]. The disease is caused by germline mutations in the VHL tumor suppressor gene on a short arm of chromosome 3. In most cases, VHL disease is caused by single base substitutions, microdeletions or microinsertions, however approximately 30% of VHL patients carry a deletion of a part or of the entire *VHL* gene [4-6].

Patients with small intragenic mutations of the *VHL* gene have a typical disease presentation. However this may not be the truth in cases of carriers of large deletions. Large deletions may involve other genes in the proximity of the *VHL* gene, i.e. *FANCD2, C3orf10, CNTN6, IRAK2, GHRL or MLH1* genes that may have an effect on the disease presentation. For example, there is some evidence to suggest that large deletions of *VHL* and of *C3orf10* are associated with lower life time risk of kidney cancer compared to the deletions which do not include the *C3orf10* gene [7-10]. However, the impact of the extent of large deletions encompassing *VHL* and other adjacent genes on phenotype has not been extensively studied. In particular, it was not investigated whether large *VHL* deletions that include the *FANCD2* gene - an important gene in the DNA damage repair signaling pathway, have an effect on disease phenotype.

## Material and methods

### Patients

**Figure 1:**
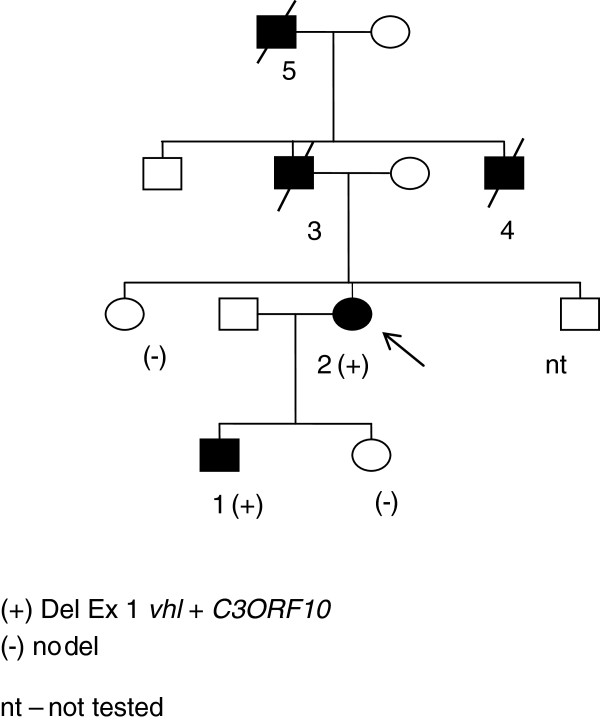
Pedegree of the affected family.

We studied a phenotype of a family with a large deletion of the *VHL* gene that involves the *FANCD2* gene. This family included 11 family members (Figure 1), 4 patients had VHL-related tumors, and 2 patients had other tumors (breast cancer, prostate cancer) (Table 1). Blood samples were available from 3 family members (a 53 year old woman with bilateral breast cancer and VHL disease, her 31 year old son with VHL disease and her healthy 30 year old daughter).

**Table 1:** VHL related and non-VHL related lesions detected in the affected family

**Patient no.**	**VHL related CNS lesions**	**VHL related visceral lesions**	**Non VHL related tumors**
1	HbCb age 18, Hbs age 19,Hbs age 31	Renal cysts	
2	HbCb –age 54	RCC age 54	BrCa – age 46
			BrCa – age 49
3	HbCb 65	NA	
4	NA	NA	Pr – age 65
5	HbCb 69	NA	

RCC renal cell carcinoma, BrCa – breast carcinoma, HbCb – heamangioblastoma of cerebellum, Hbs – spinal haemangioblastoma, Pr – prostate cancer, NA- no examination, no imaging studies.

### Mutation analysis

DNA was extracted from whole blood using standard techniques. Peripheral blood leukocytes obtained from the patient were processed for DNA isolation using standard methods. The entire coding region of *VHL* gene was sequenced using direct Sanger sequencing as described previously [6]. Large genomic rearrangements were detected using MLPA analysis using the SALSA MLPA kit P016 VHL assay (MRC Holland, Amsterdam, The Netherlands). This assay includes 17 probes from distal chromosome 3p25 (from telomere to centromere): one probe for CNTN6, two probes for *FANCD2,* two probes for *C3ORF10*, four probes for *VHL* exon 1, three probes for *VHL* exon 2, two probes for *VHL* exon 3, *IRAK2* probe, *GHR* probe, and *MLH1* probe. The products of MLPA reactions were analyzed with automated sequencer (model 373A, ABI, USA).

## Results

In two patients with VHL disease, a large germline deletion involving the exon 1 of the *VHL* gene, the *C3ORF10* gene and a fragment of the *FANCD2* gene was detected. The presence of the deletion was confirmed using MLPA in repeated analyses using DNA samples isolated from two different blood samples for each patient. In brief, in MLPA, the peak height was significantly lower for the six probes (three probes for *VHL* exon 1, two probes for *C3ORF10* and 1 probe for *FANCD2* gene) indicating the presence of a heterozygous deletion. Based of the localization of these six probes, we estimate that the length of this deletion is approximately 50 kb (between 43 kb to 54 kb). The telomeric breakpoint lies within the *FANCD2* gene between exon 35 and exon 43 of the *FANCD2* gene (a signal for the probe specific for exon 43 of the *FANCD2* gene was abnormal, and signal for the probe for exon 35 of the *FANCD2* gene was appropriate). The centromeric breakpoint is located in intron 1 the *VHL* gene.

The clinical presentation of the VHL disease in this family includes CNS heamangioblastoma seen in four patients, kidney cancer diagnosed in one patient and no retina heamangioblastoma or pheochromocytoma. Age of diagnosis and localization of these typical VHL-associated lesions are shown in Table 1.

Interestingly, one carrier of the deletion developed bilateral breast cancer at age 46 and 49. First cancer was diagnosed at the age of 46 in her right breast. The patient was treated by mastectomy with postoperative chemotherapy (CMF). Histopathological examination revealed invasive ductal breast carcinoma of 1.8 cm size. The tumor was ER- negative, and PR-negative (HER2 was not analyzed). A metastasis in 1 axillary lymph node was found. There was no evidence of distant metastases. The tumor was classified as T1c pN1a M0, stage IIA; grade G2. Three years later, at the age of 49, she was diagnosed with cancer of her left breast. It was an invasive ductal carcinoma of 2.0 cm size. The tumor was ER-positive, PR-positive, and HER2-negative. There was no evidence for lymph-node or distant metastases. The tumor was classified as T1c pN 0 M0, stage IA, grade G2 and treated with mastectomy and tamoxifen. Ki67 expression was not tested. At present, 9 and 6 years after treatment of the breast cancers there is no evidence of breast cancer recurrence. However at age of 54, lesions typical for VHL disease including cerebellar haemangioblastoma and renal cell carcinoma were diagnosed in the patient.

There was also a history of prostate cancer in one affected male family member at the age of 65 years although his DNA sample was not available for MLPA analysis.

## Discussion

To our knowledge this is the first study to suggest an association between a large deletion involving the *FANCD2* gene and bilateral breast cancer. *FANCD2* is one of eight genes known to cause the autosomal recessive disorder Fanconi anaemia (FA), which is characterized by spontaneous chromosomal instability, immunodeficiency, and a predisposition to cancer [11-14]. *FANCD2* plays an important role in the recombination DNA repair pathways [15]. The activated *FANCD2* protein co-localises with other DNA repair proteins such as *BRCA1, BRCA2, ATM, NBS1* and *RAD51*[16]. It has been suggested that *FANCD2* and *BRCA1* interact directly in this process [11]. In response to ionising radiation, *FANCD2* is also phosphorylated by ATM, which leads to the activation of an S-phase checkpoint of the cell cycle [16]. Therefore, *FANCD2* is good candidate for breast cancer susceptibility gene.

It is interesting that biallelic mutations of *BRCA2*, the major gene for hereditary breast cancer, were also shown to cause Fanconi anemia. However, it has been reported that FA gene mutations, other than in *BRCA2*, are unlikely to be a frequent cause of highly penetrant breast cancer predisposition. Analysis of the FA genes (*FANCA*, *B*, *C*, *D1*, *D2*, *E*, *F*, *G*) in 88 non-*BRCA1*, non-*BRCA2* breast cancer families failed to identify any highly penetrant mutations for breast cancer [17]. In another study, 399 women, from 356 non-*BRCA1/2* breast cancer families (some had more than one index case because multiple women were affected at the same age), were screened for *FANCD2* mutations by DHPLC/sequencing and no pathogenic mutations were identified [18].

In our study, breast cancers seen in carrier of a deletion of *FANCD2* gene were invasive, grade 2 ductal carcinomas. Both were tumors of ~2 cm in diameter. One of these had lymph node metastases. One tumor was ER- and PR-negative (HER2 status unknown) and the other was ER- and PR-positive, but HER2-negative. This may suggest that a mutation in *FANCD2* predispose to invasive breast cancer of ductal type, however further studies are needed in this regard.

Given that both *FANCD2 and BRCA2* mutations (in homozygous state) cause the same chromosomal instability syndrome called Fanconi anemia, it is likely that breast cancers that arise in carriers of a mutation in these genes may have similar clinical characteristics. In this context it is important to mention that BRCA2-associated breast cancers exhibit higher grade than sporadic tumors [19-22]. They are similar to sporadic cancers with respect to ER receptor status [20,23-25]. However, one study reported that *BRCA2 associated* tumors are more likely to be ER-positive [22]. In addition, it has been reported that *BRCA2*-related breast cancers are less likely to over express HER2 receptor compared to sporadic cancers [20,25]. Further studies are needed to investigate whether *FANCD2-*related breast cancers are similar to those seen *BRCA2* mutation carriers.

In conclusion, our study suggests that a deletion of the *VHL and FANCD2* gene may be associated with coexistence of *VHL* disease and hereditary breast cancer, but further studies are needed in this regard.

## Consent

Informed consent has been obtained from the patients for publication.

## Competing interests

The authors declare that they have no competing interests.

## Authors’ contributions

KK designed the study, collected clinical data for the study, enrolled the patients into the study group , wrote the manuscript. AJ carried out the molecular genetic studies. JG carried out the molecular genetic studies. MRK enrolled the patients into the study group. M D-S collected clinical data for the study. LS enrolled the patients into the study group. LC collected clinical data for the study. WL participated in its design and coordination. JL participated in its design and coordination. CC conceived the study, carried out the molecular genetic studies participated in writing and helped to draft the manuscript, critically revised the manuscript and approved its final version. All authors read and approved the final version of the manuscript.

## References

[B1] Maher ER Iselius L Yates JR Littler M Benjamin C Harris R Sampson J Williams A Ferguson-Smith MA Morton N von Hippel–Lindau disease: a genetic study J Med Genet 1991 28 7 443 447 1895313 10.1136/jmg.28.7.443 PMC1016952

[B2] Maher ER Yates JR Harries R Benjamin C Harris R Moore AT Ferguson-Smith MA Clinical features and natural history of von Hippel–Lindau disease Q J Med 1990 77 283 1151 1163 2274658 10.1093/qjmed/77.2.1151

[B3] Couch V Lindor NM Karnes PS Michels VV von Hippel-Lindau disease Mayo Clin Proc 2000 75 265 272 10725953 10.4065/75.3.265

[B4] Stolle C Glenn G Zbar B Humphrey JS Choyke P Walther M Pack S Hurley K Andrey C Klausner R Linehan WM Improved detection of germline mutations in the von Hippel-Lindau disease tumor suppressor gene Hum Mutat 1998 12 417 423 9829911 10.1002/(SICI)1098-1004(1998)12:6<417::AID-HUMU8>3.0.CO;2-K

[B5] Maher ER Kaelin WG Jr von Hippel-Lindau disease Medicine (Baltimore) 1997 76 381 391 9413424 10.1097/00005792-199711000-00001

[B6] Cybulski C Krzystolik K Murgia A Górski B Debniak T Jakubowska A Martella M Kurzawski G Prost M Kojder I Limon J Nowacki P Sagan L Białas B Kałuza J Zdunek M Omulecka A Jaskólski D Kostyk E Koraszewska-Matuszewska B Haus O Janiszewska H Pecold K Starzycka M Słomski R Cwirko M Sikorski A Gliniewicz B Cyryłowski L Fiszer-Maliszewska L Germline mutations in the von Hippel-Lindau (VHL) gene in patients from Poland: disease presentation in patients with deletions of the entire VHL gene J Med Genet 2002 39 7 E38 No abstract available 12114495 10.1136/jmg.39.7.e38 PMC1735187

[B7] Maranchie JK Afonso A Albert PS Kalyandrug S Phillips JL Zhou S Peterson J Ghadimi BM Hurley K Riss J Vasselli JR Ried T Zbar B Choyke P Walther MM Klausner RD Linehan WM Solid renal tumor severity in von Hippel Lindau disease is related to germline deletion length and location Hum Mutat 2004 23 40 46 14695531 10.1002/humu.10302

[B8] Casco’n A Escobar B Montero-Conde C Rodrı’guez-Antona C Ruiz-Llorente S Osorio A Mercadillo F Leto’n R Campos JM Garcı’a-Sagredo JM Benı’tez J Malumbres M Robledo M Loss of the actin regulator HSPC300 results in clear cell renal cell carcinoma protection in Von Hippel-Lindau patients Hum Mutat 2007 28 613 621 17311301 10.1002/humu.20496

[B9] Gerlind F Birke B Hoffmann MM Markus C Christian W Jurgen K Gerd S Neumann HPH Alu-Alu recombination underlies the vast majority of large VHL germline deletions: molecular characterization and genotype–phenotype correlations in VHL patients Hum Mutat 2009 30 5 776 786 19280651 10.1002/humu.20948

[B10] McNeill A Rattenberry E Barber R Killick P MacDonald F Maher ER Genotype–phenotype correlations in VHL exon deletions Am J Med Genet Part A 2009 149A 2147 2151 19764026 10.1002/ajmg.a.33023

[B11] Meetei AR de Winter JP Medhurst AL Wallisch M Waisfisz Q van de Vrugt HJ Oostra AB Yan Z Ling C Bishop CE Hoatlin ME Joenje H Wang W A novel ubiquitin ligase is deficient in Fanconi anemia Nat Genet 2003 35 165 170 12973351 10.1038/ng1241

[B12] Bagby GC Jr Genetic basis of Fanconi anemia Curr Opin Hematol 2003 10 68 76 12483114 10.1097/00062752-200301000-00011

[B13] Grompe M D’Andrea A Fanconi anemia and DNA repair Hum Mol Genet 2001 10 2253 2259 11673408 10.1093/hmg/10.20.2253

[B14] Howlett NG Taniguchi T Olson S Cox B Waisfisz Q De Die-Smulders C Persky N Grompe M Joenje H Pals G Ikeda H Fox EA D’Andrea AD Biallelic inactivation of BRCA2 in Fanconi anemia Science 2002 297 5581 606 609 12065746 10.1126/science.1073834

[B15] Houghtaling S Timmers C Noll M Finegold MJ Jones SN Meyn MS Grompe M Epithelial cancer in Fanconi anemia complementation group D2 (Fancd2) knockout mice Genes Dev 2003 17 2021 2035 12893777 10.1101/gad.1103403 PMC196256

[B16] D’Andrea AD Grompe M The Fanconi anaemia/BRCA pathway Nat Rev Cancer 2003 3 23 34 12509764 10.1038/nrc970

[B17] Seal S Barfoot R Jayatilake H Smith P Renwick A Bascombe L McGuffog L Evans DG Eccles D Easton DF Stratton MR Rahman N Breast Cancer Susceptibility Collaboration Evaluation of Fanconi Anemia genes in familial breast cancer predisposition Cancer Res 2003 63 8596 8599 14695169

[B18] Lewis AG Flanagan J Marsh A Pupo GM Mann G Spurdle AB Lindeman GJ Visvader JE Brown MA Chenevix-Trench G Kathleen Cuningham Foundation Consortium for Research into Familial Breast Cancer. Mutation analysis of FANCD2, BRIP1/BACH1, LMO4 and SFN in familial breast cancer Breast Cancer Res 2005 7 6 R1005 R1016 16280053 10.1186/bcr1336 PMC1410737

[B19] Vargas AC Silva LD Lakhani SR The contribution of breast cancer pathology to statistical models to predict mutation risk in BRCA carriers Fam Cancer 2010 9 545 553 20577821 10.1007/s10689-010-9362-5

[B20] Lakhani SR Van De Vijver MJ Jacquemier J Anderson TJ Osin PP McGuffog L Easton DF The pathology of familial breast cancer: predictive value of immunohistochemical markers estrogen receptor, progesterone receptor, HER-2, and p53 in patients with mutations in BRCA1 and BRCA2 J Clin Oncol 2002 20 2310 2318 11981002 10.1200/JCO.2002.09.023

[B21] Phillips KA Immunophenotypic and pathologic differences between BRCA1 and BRCA2 hereditary breast cancers J Clin Oncol 2000 18 107S 112S 11060337

[B22] Bane AL Beck JC Bleiweiss I Buys SS Catalano E Daly MB Giles G Godwin AK Hibshoosh H Hopper JL John EM Layfield L Longacre T Miron A Senie R Southey MC West DW Whittemore AS Wu H Andrulis IL O'Malley FP BRCA2 mutation-associated breast cancers exhibit a distinguishing phenotype based on morphology and molecular profiles from tissue microarrays Am J Surg Pathol 2007 31 121 128 17197928 10.1097/01.pas.0000213351.49767.0f

[B23] Armes JE Trute L White D Southey MC Hammet F Tesoriero A Hutchins AM Dite GS McCredie MR Giles GG Hopper JL Venter DJ Distinct molecular pathogeneses of early-onset breast cancers in BRCA1 and BRCA2 mutation carriers: a population-based study Cancer Res 1999 59 2011 2017 10213514

[B24] Palacios J Honrado E Osorio A Cazorla A Sarrió D Barroso A Rodríguez S Cigudosa JC Diez O Alonso C Lerma E Dopazo J Rivas C Benítez J Phenotypic characterization of BRCA1 and BRCA2 tumors based in a tissue microarray study with 37 immunohistochemical markers Breast Cancer Res Treat 2005 90 5 14 15770521 10.1007/s10549-004-1536-0

[B25] Lakhani SR Reis-Filho JS Fulford L Penault-Llorca F van der Vijver M Parry S Bishop T Benitez J Rivas C Bignon YJ Chang-Claude J Hamann U Cornelisse CJ Devilee P Beckmann MW Nestle-Krämling C Daly PA Haites N Varley J Lalloo F Evans G Maugard C Meijers-Heijboer H Klijn JG Olah E Gusterson BA Pilotti S Radice P Scherneck S Sobol H Prediction of BRCA1 status in patients with breast cancer using estrogen receptor and basal phenotype Clin Cancer Res 2005 11 5175 5180 16033833 10.1158/1078-0432.CCR-04-2424

